# Progress of Cholera

**Published:** 1854-08

**Authors:** 


### EDITORIAL.

*Progress of Cholera.*

In the July No. of the Journal, we noted the progress of Cholera and Dysentery in this city up to the 31st of that month. Since that time the Cholera has altogether ceased as an *epidemic*, as the following tabular statement will show:

| | CHOLERA. | ALL DISEASES. |
|---|---|---|
| August 1, | 16 | 33 |
| " 2, | 12 | 34 |
| " 3, | 10 | 27 |
| " 4, | 10 | 22 |
| " 5, | 15 | 26 |
| " 6, | 9 | 25 |
| " 7, | 16 | 28 |
| " 8, | 14 | 29 |
| " 9, | 15 | 22 |
| " 10, | 13 | 27 |
| " 11, | 5 | 21 |
| " 12, | 5 | 21 |
| " 13, | 11 | 22 |
| " 14, | 8 | 14 |
| " 15, | 7 | 15 |
| " 16, | 9 | 30 |
| " 17, | 1 | 11 |
| " 18, | 4 | 23 |
| " 19, | 3 | 14 |
| " 20, | 2 | 10 |
| " 21, | 5 | 16 |
| " 22, | 9 | 35 |
| " 23, | 5 | 29 |
| " 24, | 5 | 18 |
| " 25, | 8 | 22 |

This gives a total of 212 deaths from Cholera in 25 days, and a total of 574 from all diseases during the same time; being only little more than one-half of the mortality which took place during the preceding month. During the first week in August the weather continued warm, with a moist atmosphere. From the 6th to the 10th it was cool and dry. Some rain fell on the 11th, and the 12th was very sultry and warm. From the 13th to the 17th it was uniformly clear, dry, and moderately warm. On the 18th there was a slight fall of rain; but from the 19th to the 25th it was uniformly clear, dry, and very warm; the mercury generally rising in the middle of the day to 98 and 100 degrees Fah. During this time, too, the wind was uniformly from the south or south-west. On the evening of the 25th, we had a copious shower of rain, and the wind changed to the north. By comparing these statements in reference to the weather with the table of daily mortality, it will be seen that they bear the same relation essentially as that pointed out in our previous notices. We may now safely consider the prevalence of cholera in this city at an end for this season. Sporadic cases may continue to occur for a few weeks longer, but even these will be limited to immigrants and intemperate residents chiefly. Within a few weeks past, we have had several letters, asking our method of treating cholera. None of these letters have been answered for two reasons: First, they reached us at a time when sickness was unusually prevalent in the city, leaving us not one moment of leisure from day-light to day-light again. Second, it is impossible in one brief letter to give any adequate idea of the treatment of such a disease as cholera is in its various stages. Neither have we time or space now to do more than give a brief outline of our views. We believe cholera to consist essentially in a great diminution of organic susceptibility throughout the whole capillary system, coupled with a peculiar irritability of the mucous membranes. The first leads more or less rapidly to a suspension of capillary circulation, and consequently to diminished or suspended secretion and production of animal heat. The second is manifested in the violent vomiting and purging during the active stage of the disease. These views point distinctly to *three* leading therapeutic indications, viz.: First, to soothe the irritation of the mucous surfaces. Second, to improve the organic susceptibility and tonicity of the whole system; and third to restore the secretions of the more important organs and replenish the blood. In the first or premonitory stage of the disease, the prompt fulfilment of the two first indications is all that is required. For this purpose I have found nothing better than some one of the following formula, viz.:

| | | |
|---|---|---|
| ℞ | Chloride of Sodium - - - - | ʒij |
| | Tinct. Opii - - - - - - | ʒij |
| | Water - - - - - - - | ℥ij |

Mix and give one tea-spoonfull every 2, 3, or 4 hours until the bowels become natural.

| | | |
|---|---|---|
| ℞ | Aromatic Sulph. Acid - - | ℥j |
| | Sulph. of Quinine - - | 10 grs |
| | Sulph. of Morph. - - - | 5 grs |

Mix—give 15 or 20 drops every 4 or 6 hours in sweetened water. If in addition to either of the above remedies, we give each morning and evening a powder composed of Sub. Murias. Hydrarg. 2grs. and Pulv. Opii from half a grain to one grain, it will seldom fail in two days not only to remove the premonitory symptoms of cholera, but also invigorate the system. and remove the tendency of relapse. If more active serous or cholera diarrhoea has commenced, and yet without vomiting or cramps, we give the following powder every one or two hours until the discharges entirely cease, viz:

℞ Sub. Murias Hydrarg from 2 to 4 grs.

Acetas Plumbi from 1 to 2 grs.

Pulv. Opii from ½ to 2 grs. mixed.

At the same time keep the patient entirely at rest and give for nourishment sweet milk boiled with a little flour, or chicken tea well salted. After the bowels have been kept quiet from 18 to 24 hours, we usually give the following viz.:

| | | |
|---|---|---|
| ℞ | Tinct. Cinchonae - | ℥ij |
| | Aromat. Syrup Rhei - | ℥j |

Mix and give a tea-spoonful every 4 or 6 hours, until the bowels become regular. Sometimes it is necessary to add Tinct. Opii et Camp. ℥j. to the last prescription. In the second or active stage of cholera, when vomiting and purging, and cramps have fairly commenced, we give one of the powders of Calomel, Opium and Acetate of lead, named above, immediately after every turn of vomiting, whether it be once in five minutes or once an hour. At the same time we direct an Enema consisting of half a tea-cupful of thin starch and 10 grs. of Acetate of lead with a tea-spoonful of Laudanum, to be thrown into the rectum immediately after each evacuation of the bowels. We cover the epigastric region and central part of the spine with a strong mustard plaster, and persuade the patient if possible to lie still with the lower extremities covered. We satisfy the craving for drink as much as possible with small pieces of ice, and allow every half hour a wine-glass ful lof a weak solution of salt. Such are the chief means with which we combat cholera in its active stage; and quite as much depends on the *manner* of using them as on the means themselves. For instance, if we prescribe a powder to be given every half hour or hour, or if we wait, as is often done, a few minutes after each turn of vomiting "*for the stomach to get settled,*" the few minutes we wait is often just sufficient to have the stomach prepared for another paroxysm of vomiting; and of course the medicine is immediately rejected. But the same medicine given *immediately* after the stomach has exhausted itself will be retained long enough to *gain* some effect. The same rule is equally important in reference to enemas. In a great majority of cases the skillful application of the foregoing means, commenced in the early part of the active stage will promptly control the disease. If the active stage is more advanced, the tongue and lips cold; the pulse feeble; the voice husky, &c., we add to the above remedies one or two tablespoonfulls of chicken or beef tea thoroughly salted every 30 minutes, attended with the same quantity of strong coffee. At the same time we lessen the quantity, or entirely exclude the Opium from the powders and supply its place with from 2 to 4 grs. of Quinine.

If the patient passes into entire *collapse*, we trust chiefly to the following drinks, and entire quiet, viz.: strong infusion of coffee, two parts; sweet milk, one part; given in doses of one, two, or three fluid ounces every half hour; and between each dose the same quantity of chicken or beef tea, nearly saturated with common salt. In this stage, the fluids of the system having become exhausted, the object is, to husband what vitality remains by rest, and the restoration of the materials previously lost from the blood and tissues, in such doses as the stomach will bear. Such is a very brief outline of our treatment of this formidable disease. It is true that we often use remedies not mentioned here. We have bled some patients, cupped others, put some in the warm bath, others in the cold bath, or wet sheets. To some we have given salt and mustard; to others salt, calomel, and quinine. Sometimes we have given a large dose of calomel; sometimes nothing but very small doses, repeated every five minutes. These have been variations to meet special indications in particular cases. To point out such special indications, &c., would require all the space there is in the present journal, and far more time than we have at command. We have given the foregoing brief outline of treatment, not from any special confidence in its importance, but to gratify our correspondents as far as possible. It will be seen that we have made no mention of brandy, cayenne pepper, carb. ammonia, ether, &c., simply because we do not use them. We have done active service through *five* cholera seasons (one in New York city and four in Chicago), and during the whole time we have not given internally *one drachm* of cayenne pepper, or *one pint* of any kind of alcoholic drink Still we have had abundant opportunities for observing the effects of both, and are satisfied that the first only irritates the sensitive surface of the stomach, and the last renders the blood more carbonaceous, and less capable of sustaining the organic actions and vital properties than without it. D.

